# Polyphenolic Composition and Hypotensive Effects of *Parastrephia quadrangularis* (Meyen) Cabrera in Rat

**DOI:** 10.3390/antiox8120591

**Published:** 2019-11-27

**Authors:** Fredi Cifuentes, Javier Palacios, Chukwuemeka R. Nwokocha, Jorge Bórquez, Mario J. Simirgiotis, Ignacio Norambuena, Mario Chiong, Adrián Paredes

**Affiliations:** 1 Laboratorio de Fisiología Experimental (EPhyL), Instituto Antofagasta (IA), Universidad de Antofagasta, 1270300 Antofagasta, Chile; fredi.cifuentes@uantof.cl; 2 Laboratorio de Bioquímica Aplicada, Facultad de Ciencias de la Salud, Universidad Arturo Prat, 1110939 Iquique, Chile; 3 Department of Basic Medical Sciences (Physiology Section), Faculty of Medical Sciences, The University of the West Indies, Mona, Kingston 7, Jamaica; chukwuemeka.nwokocha@uwimona.edu.jm; 4 Laboratorio de Productos Naturales, Departamento de Química, Universidad de Antofagasta, 1270300 Antofagasta, Chile; jorge.borquez@uantof.cl; 5 Instituto de Farmacia, Facultad de Ciencias, Universidad Austral de Chile, Valdivia 5090000, Chile; mario.simirgiotis@uach.cl; 6 Advanced Center for Chronic Diseases (ACCDiS), CEMC, Department of Biochemistry and Molecular Biology, Faculty of Chemical and Pharmaceutical Sciences, University of Chile, Santiago 8380492, Chile; ienoramb@uc.cl (I.N.); mchiong@ciq.uchile.cl (M.C.); 7 Laboratorio de Química Biológica, Instituto Antofagasta (IA), Universidad de Antofagasta, 1270300 Antofagasta, Chile

**Keywords:** *Parastrephia quadrangularis*, polyphenols, hypotension, vasodilation, antioxidant capacity

## Abstract

*Parastrephia quadrangularis* (Pq), commonly called “Tola”, is widely used in folk medicine in the Andes, including for altitude sickness. In this study, polyphenolic composition was determined, and hypotensive effects were measured; the ethnopharmacological use as hypotensive was related to the presence of phenolic compounds. For this purpose, male Sprague-Dawley rats (6 to 8 weeks of age, 160 to 190 g) were fed Pq extract (10 to 40 mg/kg) for 10 days through gavage. Blood pressures and heart rate were significantly (*p* < 0.01) reduced in normotensive rats receiving Pq extract (40 mg/kg body weight). Pq extract induced a negative inotropic effect, and endothelium-dependent vasodilation mediated by nitric oxide (NO). Furthermore, preincubation with Pq extract significantly decreased the cytosolic calcium on vascular smooth muscle cells A7r5 in response to L-phenylephrine (PE). Seven metabolites were isolated from the Pq extract, but three flavonoids (10^−4^ M) showed similar vasodilation to the extract in intact rat aorta as follows: 5,3′,4′-trihydroxy-7-methoxyflavanone (2); 3,5,4′-trihydroxy-7,8,3′-trimethoxyflavone (6); and 5,4′-dihydroxy-3,7,8,3′-tetramethoxyflavone (7). The Pq extract and compounds 2 and 7 significantly (*p* < 0.05) reduced the contraction to Bay K8644 (10 nM, an agonist of Ca_V_1.2 channels). Administration of Pq decreased cardiac contractility and increased endothelium-dependent and -independent vasodilation.

## 1. Introduction

*Parastrephia quadrangularis* (Meyen) Cabrera, is a native shrub belonging to the *Asteraceae* family, found in the Northern Andes of Chile, Argentina, Southern Perú, and Bolivia. The plant heights are in the range of 0.3–1.5 m and several plants grow together forming a “piso puneño” or “tolar”, a green spot in the desert between 3500 and 4200 m above the sea level. for cattle feeding with great economic significance [1,2]. This plant is also medicinal and its infusions are widely used in the Andes, since Aymara aboriginal times, to treat fever, inflammatory conditions, and altitude sickness [1,3]. It is also used to counteract urinary infections and respiratory diseases [4].

The *Parastrephia* genera has been shown to possess significant biological activities such as inhibition of the cyclooxygenase enzymes COX-1 and COX-2 [5], inhibition of arachidonic acid [6], and inhibition of proinflammatory enzymes [7], as well as the *Parastrephia* genera have been reported to have antimicrobial and antifungal capacities [8,9,10], plus antiproliferative [11], and photoprotective activities [12]. The tremetones isolated from *Parastrephia lepidophylla* (Wedd.) Cabrera and *Parastrephia lucida* (Meyen) Cabrera showed analgesic and antioxidant activities [13,14]. In addition, Pq showed protective activity against oxidative damage in human erythrocytes [15], and significant antifungal activity [16]. 

From the phytochemical point of view, some bioactive metabolites have been isolated from Pq which include: 5,7-dihydroxy-3,3′,4′,8-tetramethoxyflavone; p-cumaroyloxytremetone; coumaric acid; and kaempferol [15,17]; whereas two compounds were only tentatively identified by means of low-resolution mass spectrometry which include: coumaroyloxytremetone-hexoside and coumaroyloxytremetone-C-hexoside [16].

Our study represents the first work to elucidate polyphenolic composition and the cardiovascular effects of Pq in an animal model. The extracts and metabolites isolated were studied with regards to their effects on arterial blood pressure, as well as the cardiac and vascular tissues. The antioxidant capacities of the Pq were also evaluated using several in vitro assays. 

## 2. Material and Methods 

### 2.1. Drugs

The drugs used were L-phenylephrine hydrochloride (PE); acetylcholine chloride (ACh); 1H-(1,2,4)oxadiazolo[4,3-a]quinoxalin-1-one (ODQ); Nω-nitro-L-arginine methyl ester (L-NAME); and (±)-Bay K8644 (Sigma-Aldrich, St Luis, MO, USA). Nimodipine, tetraethyl ammonium (TEA), barium chloride dihydrate (BaCl_2_), glibenclamide, and quercetin were obtained from Merck (Darmstadt, Germany). Several drugs were dissolved in distilled deionized water (deionized water Millipore) and kept at 4 °C. The stock solution of ODQ, glibenclamide, quercetin, nimodipine, and (±)-Bay K8644 was dissolved in dimethyl sulfoxide (DMSO, 0.1% final concentration) (Merck, Darmstadt, Germany). Physiological Krebs-Ringer bicarbonate (KRB) containing (mM): 4.2 KCl, 1.19 KH_2_PO_4_, 120 NaCl, 25 NaHCO_3_, 1.2 MgSO_4_, 1.3 CaCl_2_, and 5 D-glucose (pH 7.4) was used in all vascular experiments.

### 2.2. Plant Material

The plant material (branches, leaves, and inflorescences) from *P. quadrangularis* was collected from the Antofagasta region of Chile (22°19′31.80′′ S y 68°00′22.20′′ W, at 4000 m above the sea level, November 2015), and was subsequently identified and stored with a voucher number: PQ20151115.

### 2.3. Extract Preparation

The specimens were then dried and mechanically grounded to fine powder to exhaustively extract the principles to use in the pharmacological study (all procedures performed at room temperature 25 °C). A mass of 1.5 kg of the dry and powdered plant was deposited into a cotton bag with 3 L of a mixture EtOH:H_2_O (1:1) for 72 h inside a glass beaker at room temperature. Then, Whatman (filter paper) was used to filter the resulting solution; a rotary evaporator (50 °C) was subsequently used to evaporate the ethanol. The resulting aqueous extract was freeze-dried with a Labconco 4.5 FreeZone lyophilizer. The total extract yield was about 26%, which was then stored at 4 °C.

### 2.4. Extraction and Isolation of Secondary Compounds

The hydroalcoholic extract was resuspended in distilled water and extracted successively with chloroform and ethyl acetate. The organic solutions were concentrated on a rotary evaporator and lyophilized. The chloroform sub-extract (20 g) was subjected to successive steps using open column chromatography in order to isolate the bioactive constituents. The columns were packed with silica gel 60, and the chromatography was developed using solvents of increasing polarity (mixtures of *n*-hexane:EtOAc 6:4 to 1:9 *v:v*) and CHCl_3_-MeOH (1:9 to 4:6 *v:v*). We used Kiesegel F254 Thin Layer Chromatography (TLC) plates to perform TLC analyses and n-hexane:EtOAc 8:2 *v/v* to develop the plates. To visualize the spots, a mixture of 2% sulphuric acid in ethanol was sprayed on the plates. The column afforded seven sub-fractions (Pq-1 to Pq-7), which were pooled in four fractions according to TLC analysis. From fraction Pq-4, 2 g, thorough Sephadex LH-20 permeation (2 cm × 50 cm) using methanol solvent, the following known flavones were obtained: 5,4′-dihydroxy-7,3′-dimethoxyflavanone, 57.2 mg (1) [18] and 5,3′,4′-trihydroxy-7-methoxyflavanone (7-methoxy-eriodictyol), 47.2 mg (2) [19]. From fraction Pq-3, 2.7 g, the coumarin scopoletin (7-hydroxy-6-methoxy-2H-1-benzopyran-2-one, 11.5 mg (3) [20] plus the flavones 3,5,4′-trihydroxy-7,8,3′-trimethoxyflavone, 12.3 mg (6) and 5,4′-dihydroxy-3,7,8,3′-tetramethoxyflavone, (ternatin), 23.2 mg (7) [21] were obtained. From fraction Pq-2, 1.5 g, the compound p-cumaroyloxytremetone, 58 mg (4) [13] plus 18.3 mg of 5-hydroxy-7,3′,4′-trimethoxyflavanone (5) were isolated. The isolated metabolites were repurified by successive crystallization steps using different solvent mixtures of ethyl acetate and n-hexane at low temperature to assure purity (95% to 98% by High Performance Liquid Chromatography, HPLC).

### 2.5. UHPLC-DAD-MS Instrument

A Thermo RS 3000 Q exactive focus was used as an System Ultra-High Performance Liquid Chromatography-Mass Spectrometry (UHPLC-MS; Thermo Fisher Scientific, Bremen, Germany), following methods as described by Simirgiotis [22]. Briefly, 10 μL of mixture (containing 2 mL methanol and 5 mg of our extract) was injected into the instrument following filtration using Polytetrafluoroethylene (PTFE) 200 μm filters.

UHPLC-MS Solvents were obtained from Merck in Santiago Chile (formalin, reagent grade chloroform, LC-MS formic acid, deuterated chloroform, reagent grade lansoprazole, HCl, ethanol, and deuterated methanol). Other high purity agents (95%) were obtained from Extrasynthèse (Genay, Lyon, France), ChromaDex (Santa Ana, CA, USA), and Sigma-Aldrich (Saint Louis, MI, USA)

### 2.6. LC and MS Parameters

An Acclaim UHPLC C18 column at 25 °C Thermo Scientific equipment manufactured in Bremen, Germany) (150 mm × 4.6 mm ID, 2.5 μm, was used for the analysis. The wavelengths were set at 330, 254, 354, and 280 nm, and the photodiode array detector was used from 800 to 200 nm. Aqueous and mobile phases were observed 1% formic acid solutions. The gradient employed was: 0.00 min, 5% B; (5.00 min, 5% B; 10.00 min, 30% B; 15.00 min, 30% B; 20.00 min, 70% B; 25.00 min, 70% B; 35.00 min, 5% B) and lastly, before each injection (at volume 10 μL), a waiting time of 12 min for equilibration, at 1.00 mL min^−1^ flow rate. The resin extract and standard compounds were incubated at 10 °C in the autosampler before the injections. Detection of all compounds was performed using a Q-Exactive Orbitrap mass spectrometer at 17,500 Full Width at Half Maximum (FWHM) (*m/z* 200), and the HESI II probe values were optimized as previously described [22,23].

### 2.7. Animals

To evaluate the traditional use of the plant, 37 male Sprague-Dawley rats (6 to 8 weeks of age, 160 to 190 g) from the Antofagasta University breeding colony were randomly assigned into the following groups: Group 1 (*n* = 5) was used for measurement of blood pressure after intravenous bolus of Pq. In this group, Pq extract was acutely injected (intravenously) in cumulative doses (10 to 80 mg/kg) in the same rats after recovery of basal pressure. Groups 2 to 5 received gavage, vehicle or Pq extract. Group 2 was used as control, and only received vehicle (saline solution). Groups 3 to 5 (*n* = 5) were used for measurement of blood pressure after oral treatment with Pq for 10 days (10, 20, and 40 mg/kg body weight Pq). Group 6 (*n* = 7) for Langendorff was used for preparation and group 7 (*n* = 5) was used for vascular reactivity experiments. Animals had access to food (standard rat chow from Champion, Santiago, Chile) and water ad libitum, in a temperature and light controlled room. Experiments were in accordance to institutional (Universidad Antofagasta Ethics Committee, CEIC 135/2018) and use of laboratory animal care (National Institutes of Health, revised 2013).2.8. Measurement of Blood Pressure.

Blood pressure was measured on the rats with procedures previously described [24]. The rats were anesthetized with xylazine (5 mg/kg, i.p.) and ketamine (42 mg/kg, i.p.). Following the method as described above, we measured blood pressure in vivo using a pressure transducer TSD 120 connected to a DA100B amplifier (Biopac Systems Inc, Santa Barbara, CA USA). AcqKnowledge III systems software v3.9.1.6 (Santa Barbara, CA, USA) was used for blood pressure recording and data analysis.

### 2.8. Langendorff

Male Sprague-Dawley rats were anesthetized with ketamine (90 mg/kg, i.p.) and xylazine (10 mg/kg, i.p.). After (800 International Units kg, i.p.) heparinization for 5 min, the heart was separated and mounted in the Langendorff setup. A polyvinyl chloride balloon was inserted into the left ventricle and was used to determined contractile function. A total of 10 mL/min of Krebs-Henseleit buffer (KHB) containing (in mM): 4.7 KCl, 1.2 KH_2_PO_4_, 118 NaCl, 25 NaHCO_3_, 1.2 MgSO_4_, 1.75 CaCl_2_, 0.5 EDTA, and 8 D-glucose (pH 7.4, 37 °C, 95% O_2_, and 5% CO_2_) was used as a constant perfusate, then stabilization for 10 min. The balloon was filled with saline (0.9% NaCl), with end diastolic pressures between 4 and 10 mmHg. Using a Grass S-88X (Astro-Med, Inc., West Warwick, RI, USA) electrical stimulator, heart rate was fixed at 360 bpm, with intensity of 5 V for duration of 1 ms, and frequency of 6 Hz. PowerLab8 system (ADInstruments, Castle Hill, Australia) was used to record the data. HR left ventricular pressure and first derivative of intraventricular pressure (dP/dtmax and dP/dtmin) were continuously registered using the Chart for Windows 4.2. Pq extracts (1, 10, 100, 1000 µg/mL) were administered after dilutions in KHB.

### 2.9. Isolation of Aortic Rings

Following the sacrifice of the animal by cervical dislocation, the aorta was separated and transferred to a Krebs-Ringer bicarbonate buffer (KRB) solution (4 °C) (mM): 4.2 KCl, 1.19 KH_2_PO_4_, 120 NaCl, 25 NaHCO_3_, 1.2 MgSO_4_, 1.3 CaCl_2_, and 5 D-glucose (pH 7.4). Then, 3 to 4 mm rings were prepared, and cleaned of connective tissue, taking special care to avoid endothelial damage.

### 2.10. Vascular Reactivity Experiments

Aortic rings from the same animal were concurrently studied in different organ baths [25] for comparable abilities and reactivity function. After a 30 min period of equilibration, the aortic rings were stabilized with KCl (mM) near-maximum contractions for 10 min. We maintained a passive tension of 1.0 g on the aorta, which was determined to be the optimal resting tension for obtaining maximum active tension [26] in our laboratory.

Vasodilation to 10^−5^ M ACh (muscarinic agonist) in aortic rings pre-contracted with 10^−6^ M PE was used as a method to assess endothelial function. Functionality was confirmed with a vasodilation of 70% to 80% [27]. Vascular reactivity of the extracts was observed with the addition of pure and crude compound dilutions into the organ bath following rings pre-contracted with 10^−6^ M PE, or preincubation with Pq for 20 min, then contraction with 10^−6^ M PE.

In some experiments, a similar protocol was repeated in the absence of endothelium (endothelial removal was performed by gently rubbing the inner lining using a small piece of cotton), or in presence of N_w_-nitro-L-arginine methyl ester (L-NAME, 10^−4^ M).

### 2.11. Cytosolic Calcium Signal on Vascular Smooth Muscle Cells

Vascular smooth muscle cell line A7r5 (ATCC CRL-1444) were cultured in cover slips and treated with 10 µM Fluo-3 AM (Thermo Fisher Scientific Waltham, MA, USA) in KRB for 30 min at 37 °C. A Carl Zeiss LSM-5 Pascal 5 fluorescence Axiovert 200 microscope was used to study the cells at 527 nm. Cells were pretreated for 20 min with Pq (100 µg/mL) or vehicle, and then stimulated with PE 10^−6^ M. Images were collected every 1 s and analyzed with ImageJ software (v1.8.0_112, Bethesda, MD, USA, NIH). Cytosolic Ca^2+^ is expressed as ΔF/F_0_ (relative fluorescence).

### 2.12. Determination of Antioxidant Activity

In vitro, antioxidant activity was determined using the methods described by Larrazabal-Fuentes et al. 2019 [28] in Supplementary File 1 (Supplementary Materials). The absorbance of each assay was determined in a microplate reader (BioTek Synergy HTX Multimodal equipment; Winooski, VT, USA).

### 2.13. Statistical Analysis

Data were expressed as average ± standard error (SEM). A Bonferroni post hoc test was performed following a two-way analysis of variance (ANOVA) between dose-response curves. IC_50_ was calculated by nonlinear regression (sigmoidal) and *p* < 0.05 was considered statistically significant. Graph Pad PrismTM software, version 5.0. (GraphPad Software, Inc., La Jolla, CA, USA) was used.

## 3. Results

### 3.1. The Hydroalcoholic Extract from P. quadrangularis Causes a Hypotensive Effect in Rats

To evaluate the hypotensive effect of extract on blood pressure, we intravenously injected a bolus of different doses of Pq (10 to 80 mg/kg bw).

**Figure 1:**
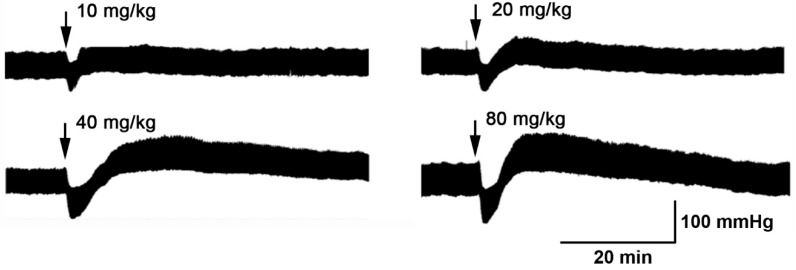
Original trace showing the hypotensive effect of *P. quadrangularis* (Pq) on the blood pressure. Pq (10, 20, 40, and 80 mg/kg bw) were administrated intravenously in normotensive rats.

It is common for hypotensive substances to cause a rapid vasodilator effect, decrease total peripheral resistance, and a compensatory effect of the cardiovascular system recovers normal blood pressure through increased cardiac output and heart rate, as a reflex effect (Figure 1).

Thus, we found that Pq extract caused a dose-dependent reduction in the mean arterial pressure (MAP) of normotensive rats (Table 1).

**Figure 2:**
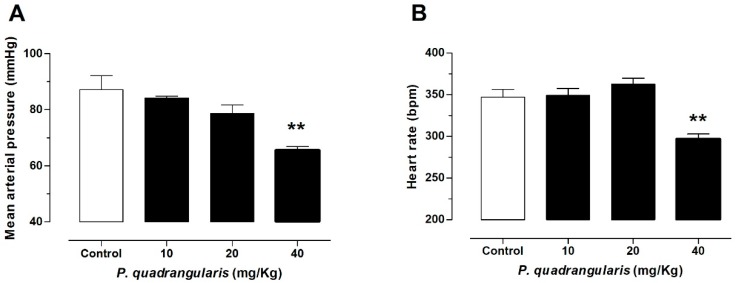
Hypotensive effects of oral administration of *P. quadrangularis* in normotensive rats. The animals were treated with 10, 20, and 40, mg/kg bw Pq for 10 days. Oral administration of extract decreased significantly (** *p* < 0.01) the mean arterial pressure (**A**), and the heart rate (**B**) as compared with control animals. Values are mean ± standard error of the mean of 5 to 7 experiments in mmHg.

In the following experiments, doses between 10 and 40 mg/kg of the extract were administered through a gastric gavage for 10 days. Pq extract significantly reduced the mean arterial pressure (87 ± 5 mmHg control vs. 66 ± 1 mmHg with 40 mg/kg Pq, *p* < 0.01, *n* = 5, Figure 2A), the heart rate (347 ± 9 bpm control vs. 297 ± 6 bpm with 40 mg/kg Pq, *p* < 0.01, Figure 2B), and the diastolic blood pressure (DBP) in normotensive rats (75 ± 6 mmHg control vs. 49 ± 1 mmHg with 40 mg/kg Pq, *p* < 0.01, Table 2).

**Figure 3:**
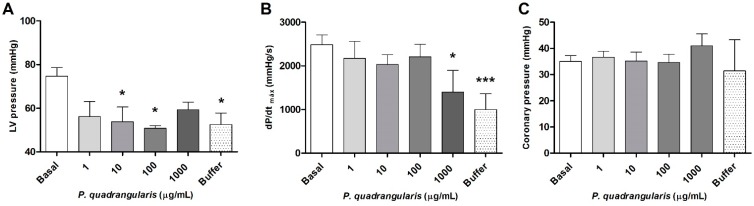
Effects of *P. quadrangularis* (Pq) on the left ventricular (LV) pressure (**A**) and the ventricular contractility (dP/dt_max_) in the Langerdorff setup (**B**). We did not observe any changes on the coronary pressure (**C**) in presence of Pq. Values are mean ± standard error of the mean of 7 experiments. * *p* < 0.05 and *** *p* < 0.001 versus basal.

To study the effect of Pq extract on cardiac contractility, Langendorff was used. We confirmed that perfusion of the heart with the extract caused a dose-dependent negative inotropic effect. Although the maximal rate of increase (dP/dt_max_) of left ventricular pressure did not decrease significantly with 100 µg/mL of the extract (Figure 3B), the left ventricular pressure (LV pressure) was drastically reduced with 100 µg/mL of the extract (75 ± 4 mmHg basal vs. 51 ± 1 mmHg with 100 µg/mL Pq, *p* < 0.05, Figure 3A). The dose of 1000 µg/mL also reduced (*p* < 0.05) the contractility (dP/dt_max_). The coronary perfusion pressure was stable in the presence of increasing doses of the extract (Figure 3C). Perfusion of the isolated heart with KHB buffer did not recover the baseline contractility value (dP/dt_max_), nor the left ventricular pressure, suggesting that effect of Pq remains. This result of washing with buffer was similar to that published with *Senesio nutans* or *Xenophylum popusum* [24,29].

### 3.2. The Hydroalcoholic Extract from P. quadrangularis (Pq) Induces an Endothelial-Dependent Vasodilator Effect in Rat Aorta

**Figure 4:**
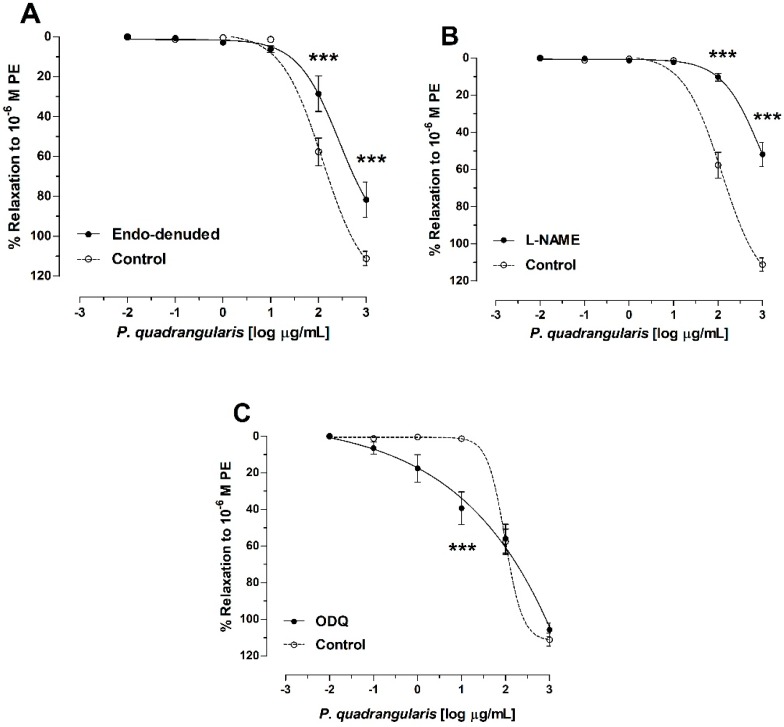
Relaxation effect of *P. quadrangularis* (Pq) in rat aorta. Concentration-response curves for *P. quadrangularis* (Pq) in endothelium intact (control) and endothelium denuded (endo-denuded) aortic rings (**A**), in presence of 10^−4^ M Nω-nitro-L-arginine methyl ester (L-NAME); (**B**), and 10^−4^ M 1H-(1,2,4)oxadiazolo[4,3-a]quinoxalin-1-one (ODQ) in the intact aorta (**C**). Data are the average ± SEM of 5 independent experiments. *** *p* < 0.001 vs. control (intact aorta).

Figure 4 shows the vasodilation dose-response curves in endothelium denuded and intact rings, or rings preincubated with L-NAME for the Pq-induced relaxation (−3 to 3 [log µg/mL], which is equivalent to 0.001 to 1000 µg/mL) As shown in Figure 4A, relaxation was lower in endothelium-denuded aortic rings in the presence of Pq extract (58 ± 7% in control vs. 29 ± 9% in endothelium-denuded aorta, 2 [log µg/mL] or 100 µg/mL, *p* < 0.001, *n* = 5). To understand the role of NO in mechanisms associated with Pq activity, we employed the use of endothelial pharmacological modulators.

The inhibition of nitric oxide synthase (NOS) with 10^−4^ M L-NAME significantly (*p* < 0.001) blunted the relaxation of Pq. However, 1H-(1,2,4) oxadiazolo[4,3-a]quinoxalin-1-one (ODQ, an inhibitor of soluble guanylyl cyclase) significantly increased (*p* < 0.001) the Pq-induced vasodilation in aortic rings versus control (Figure 4C). The IC_50_ to Pq was significantly raised (*p* < 0.001) in the absence of endothelium and in the presence of L-NAME, while the preincubation with ODQ significantly (*p* < 0.001) decreased the IC_50_ to Pq versus control (Table 3).

### 3.3. Role of Potassium Channels on Vasodilation of P. quadrangularis (Pq)

**Figure 5:**
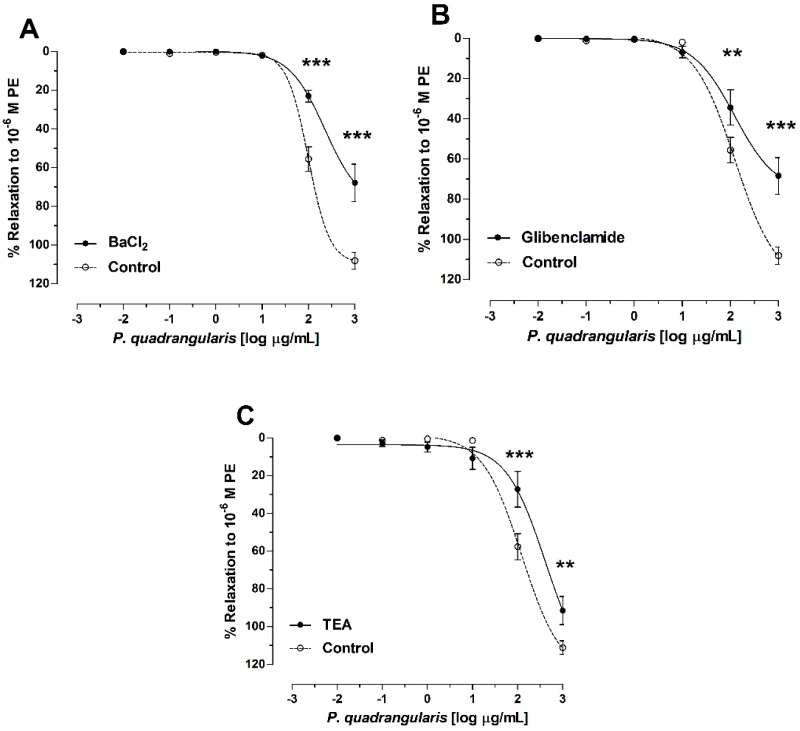
Evaluation of the *P. quadrangularis* (Pq) vasodilatation mechanism associated with potassium channels. Effect of Pq after the addition of BaCl_2_ (10^−5^ M) as a nonselective blocker of inward rectifier potassium channels (Kir) is shown (**A**), glibenclamide (10^−5^ M) as blocker of Adenosine triphosphate (ATP)-sensitive K^+^ channels (**B**), and TEA (10^−3^ M) as a nonselective blocker of KCa1.1 channels (**C**). L-phenylephrine hydrochloride (PE) (10^−6^ M) was used to induce the contractile responses to the aortic rings. Data are the average ± SEM of 5 independent experiments. ** *p* < 0.01 and *** *p* < 0.001 vs. control.

At Pq extract concentrations of 2 [log µg/mL] or 100 µg/mL and 3 [log µg/mL] or 1000 µg/mL, there were significant differences with respect to the control, where relaxation was lower in the presence of the 10^−5^ M BaCl_2_, 10^−5^ M glibenclamide, and 1 mM tetraethylammonium (TEA, *p* < 0.001, Figure 5). The IC_50_ to Pq was significantly (*p* < 0.001) raised in the presence of BaCl_2_, and TEA versus control (Table 3 and Figure 5).

### 3.4. P. quadrangularis (Pq) Reduced the Contractile Response to KCl and PE

**Figure 6:**
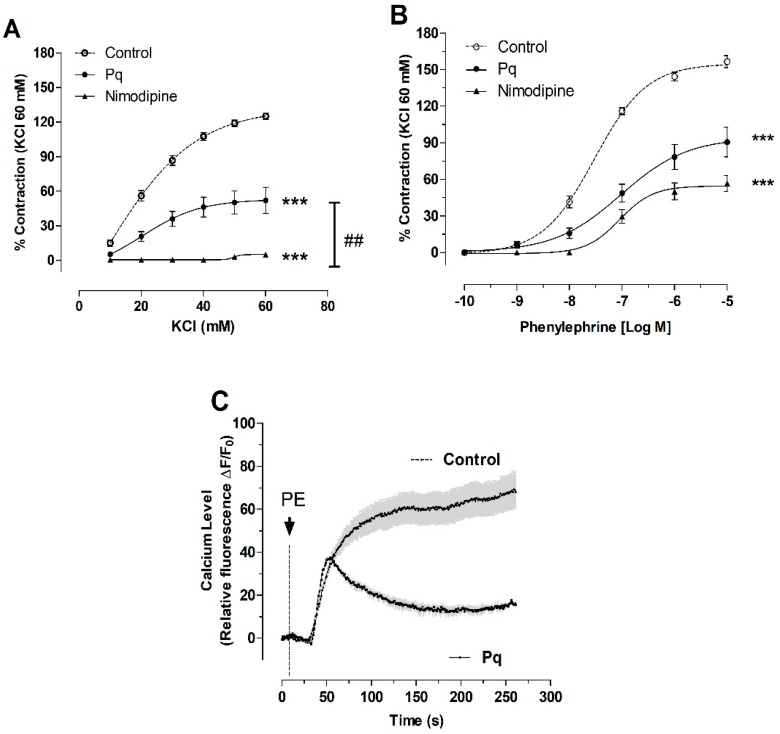
Dose-response curves of the effect of *P. quadrangularis* (Pq) on KCl (**A**), and phenylephrine (**B**) contractile response in aortic rings of rat. The cytosolic Ca^2+^ signal was stimulated with 10^−6^ M phenylephrine (PE) in the vascular smooth muscle cell line A7r5 (**C**). The aorta and cells were preincubated in the absence (control) or the presence of Pq (100 μg/mL) or nimodipine (10^−4^ M) for 20 min. Values are the mean ± standard error of the means of 3 to 5 experiments. Statistically significant differences *** *p* < 0.001 vs. control, ## *p* < 0.01 vs. Pq.

**Figure 7:**
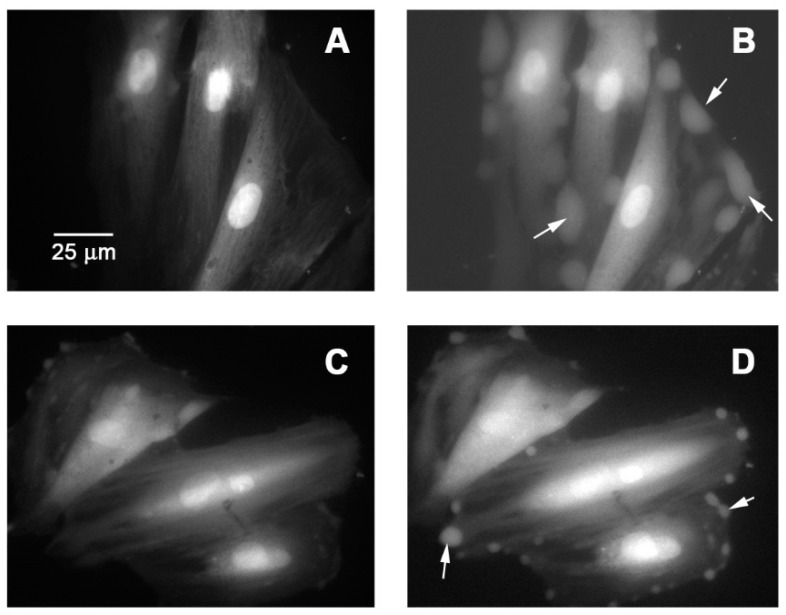
Micrograph of vascular smooth muscle cell line A7r5 stimulated with phenylephrine (PE, 10^−6^ M) (**B**) versus basal (**A**), and preincubated with *P. quadrangularis* (100 μg/mL) for 20 min and stimulated with PE (**D**) versus basal (**C**). The white arrows indicate the increase in cytosolic Ca^2+^.

The effect of Pq extract on contractile response to KCl and PE in aortic rings of the rat was studied. The preincubation with Pq extract (100 µg/mL) significantly (*p* < 0.001) reduced the maximal contraction to 60 mM KCl (125 ± 3% control vs. 52 ± 11%, Figure 6A), and the maximal contraction in response to 10^−6^ M PE (145 ± 4% for control vs. 85 ± 8%, with 100 µg/mL of Pq, *p* < 0.001). The EC_50_ to PE was significantly (*p* < 0.01) increased in the presence of Pq extract versus control (Table 4). Nimodipine was used to compare if Pq extract is a blocker of L-type voltage-gated Ca^2+^ channels. In this sense, 10^−4^ M nimodipine significantly (*p* < 0.001) reduced the maximal contraction to 60 mM KCl (125 ± 3% control vs. 5 ± 2% with nimodipine, Figure 6A) and 10^−6^ M PE (156 ± 5% control vs. 57 ± 7% with nimodipine, Figure 6B). Interestingly, with 100 µg/mL Pq preincubation, there was a decrease in the cytosolic calcium signal on vascular smooth muscle cell line A7r5 response to 10^−6^ M PE stimulation (Figure 6C and Figure 7).

### 3.5. Isolation and Structural Elucidation of Compounds

**Figure 8:**
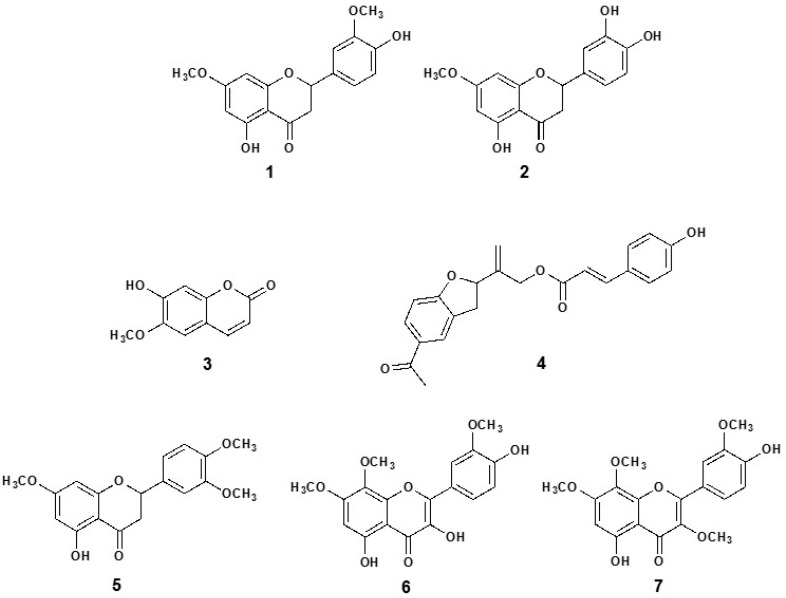
Chemical structure of the pure compounds identified from *P. quadrangularis* (Pq): 5,4′-dihydroxy-7,3′-dimethoxyflavanone (**1**); 5,3′,4′-trihydroxy-7-methoxyflavanone (7-methoxy-eriodictyol) (**2**); 7-hydroxy-6-methoxy-2H-1-benzopyran-2-one, (scopoletin, **3**); p-coumaroyloxytremetone, (**4**); 5-hydroxy-7,3′,4′-trimethoxyflavanone (**5**); 3,5,4′-trihydroxy-7,8,3′-trimethoxyflavone (**6**); and 5,4′-dihydroxy-3,7,8,3′-tetramethoxyflavone (ternatin, **7**).

The chloroform-soluble sub-fraction of Pq was subjected to a series of open column chromatographic (CC) separations to obtain the following seven known biologically active compounds (Figure 8): 5,4′-dihydroxy-7,3′-dimethoxyflavanone (1) [18]; 5,3′,4′-trihydroxy-7-methoxyflavanone (7-methoxy-eriodictyol) (2) [19]; 7-hydroxy-6-methoxy-2H-1-benzopyran-2-one, (scopoletin, 3) [20]; p-coumaroyloxytremetone (4) [13]; 5-hydroxy-7,4′,3′-trimethoxyflavanone (5); 3,5,4′-trihydroxy-7,8,3′-trimethoxyflavone (6); and 5,4′-dihydroxy-3,7,8,3′-tetramethoxyflavone (ternatin, 7) [21]. All NMR spectra (1D and 2D NMR), and mass spectrometry (Table S1) were consistent with data reported for these known compounds [13,21,30,31,32,33].

### 3.6. Vasodilation of the Pure Compounds from P. quadrangularis (Pq)

**Figure 9:**
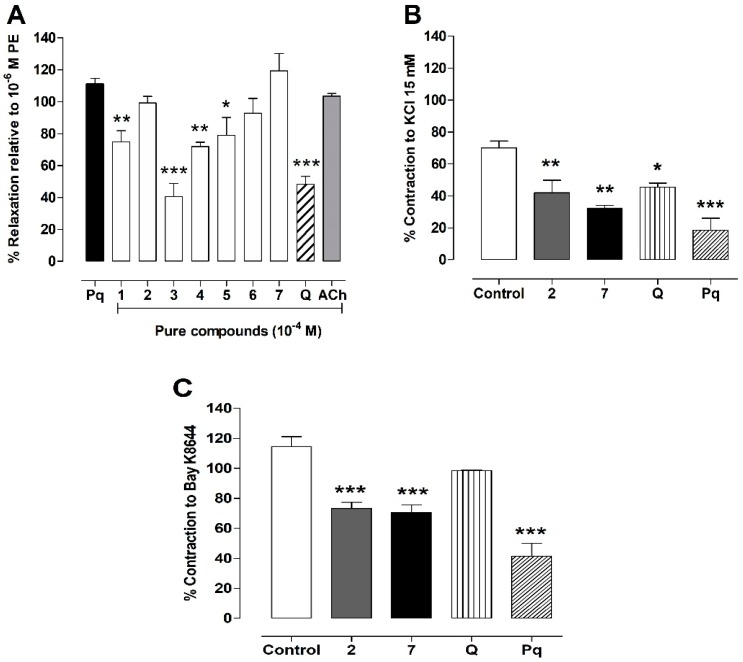
Effects of 7 pure isolated compounds from *P. quadrangularis* (Pq) on vascular response. Endothelial relaxation effect of Pq (100 μg/mL), 7 pure compounds (10^−4^ M), quercetin (Q, 10^−4^ M), and acetylcholine (Ach, 10^−4^ M) on intact aortic rings pre-constricted with 10^−6^ M phenylephrine (PE) (**A**). Vasoconstriction occurred just when the agonist of Ca_V_1.2 channels (10^−8^ M Bay K8644) was added with 15 mM KCl (**B**) to the bath (**C**). Values are mean ± SEM of 4 experiments. * *p* < 0.05, ** *p* < 0.01, ***p < 0.001 vs. Pq or control.

To provide some information on the vascular effect of the seven pure isolated compounds from Pq partition extract, we studied their effects on aortic rings pre-contracted with 10^−6^ M PE. We found that compounds 1 to 7 (Figure 9A) tested possessed some vasodilator effects. The relaxation effect of pure compounds was compared to Pq hydroalcoholic extract (100 μg/mL), in addition to quercetin and acetylcholine (10^−4^ M ACh). Interestingly, the flavonoids 2, 6, and 7 presented a high vasodilation (99 ± 4% for 2, 93 ± 9% for 6 and 119 ± 11% for 7) versus 100 μg/mL Pq extract (111 ± 4%) or 10^−4^ M acetylcholine (Ach, 104 ± 2%).

In the following experiments, we compared the relaxation and blocking of Cav1.2 channels by Pq, flavonoids 2 and 7, and quercetin. On the one hand, Pq extract, the flavonoids 2 and 7 (*p* < 0.01), and quercetin (*p* < 0.05) reduced the contractile response to 15 mM KCl (Figure 9B). On the other hand, quercetin did not decrease the contractile response to 10^−8^ M Bay K8644, an agonist of Ca_V_1.2 channels, versus control (Figure 9C).

### 3.7. Determination of the Antioxidant Content of P. quadrangularis (Pq)

Results from the quantitative determination of in vitro antioxidant activity for *Pq* are summarized in Table 5. The quantification of the content of phenolic compounds and flavonoid in Pq demonstrated the extract that contained the highest amount of polyphenols, i.e., 482 ± 19 mg gallic acid equivalent/g extract and a moderated value of flavonoids with 140 ± 4 mg quercetin equivalent/g extract, respectively. The nitric oxide radical quenching activity of the Pq was detected and compared with the standard ascorbic acid. The extract exhibited a low capacity to inhibit the nitric oxide radical, with an IC_50_ value of 498 ± 5 μg/mL in a concentration-dependent manner. Ascorbic acid inhibited the nitric oxide radical, with an IC_50_ value of 48 ± 1 μg/mL, the latter being 10 times more effective.

The ferric reducing/antioxidant power (FRAP) assay is based in the reduction of Fe^+3^ to Fe^+2^ in the presence of TPTZ (2,4,6-tris-(2-pyridyl)-s-triazine) and an antioxidant agent, thus, forming an intense complex of blue Fe-TPTZ. The FRAP assay result of the Pq shows that the extract possessed a high reducing power with 760 ± 12 mg trolox equivalent/g extract. The extract provided an antiradical activity dose-dependently inhibiting the radical 2,2-diphenyl-1-picryl-hydrazyl-hydrate (DPPH) and 2,2′-azino-bis(3-ethylbenzothiazoline-6-sulfonic acid (ABTS) with an IC_50_ value of 201 ± 4 and 127 ± 5 μg/mL, respectively. The inhibitory activity was approximately three times lower than the trolox standard for the DPPH radical with an IC_50_ value of 61 ± 3 μg/mL and two times lower for the radical ABTS, which showed IC_50_ values of 77 ± 2 μg/mL, respectively. The total antioxidant potential of the extract was estimated in phosphomolybdate and hexacyanoferrate assay, the extract exhibits a high reduction capacity with values of IC_50_ of 115 ± 4 μg/mL and 73 ± 2 μg/mL, respectively. The standard ascorbic acid shows values of 23 ± 1 μg/mL and 19 ± 3 μg/mL, in both methods studied.

### 3.8. Metabolomic Analysis of P. quadrangularis (Pq) using UHPLC-MS

**Figure 10:**
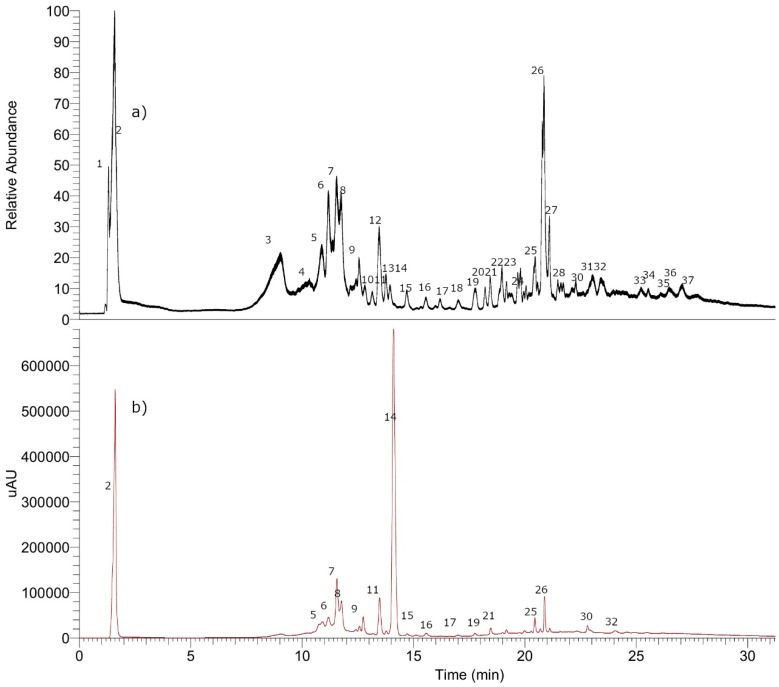
UHPLC chromatograms: (**a**) (TIC) total ion current and (**b**) UV at 280 nm of *P. quadrangularis* (Pq). Numbers of the peaks represent the metabolites. The details of metabolites are depicted in the Table S1, Supplementary Materials, plus examples of full MS spectra in Figure S1.

Thirty-seven compounds were identified including four tremetones (peaks 9, 35 to 37), three oxylipins (peaks 22, 25, and 34), twelve flavonoids (peaks 18 to 21, 23, 24, 26 to 28, and 30 to 32), nine phenolic acids (peaks 5 to 7, 11, 14 to 17, and 29) one simple organic acid (peak 1) and seven coumarins, (peaks 2 to 4, 8, 10, 12, and 13) in the chromatogram of the ethanol extract of *P. quadrangularis.* (Figure 10, Table S1 Supplementary Materials). Figure S1 (Supplementary Materials) shows spectra and structures of compounds detected as examples. The detailed identification is explained below.

#### 3.8.1. Simple Organic Acids

Peak 1 with a pseudomolecular ion at *m/z* 195.05057 was identified as gluconic acid (C_6_H_11_O_7_^−^).

#### 3.8.2. Phenolic Acids

Peaks 14 and 15 were identified as caffeoyl-quinic acid isomers (DiCOA, C_25_H_23_O_12_^−^) [34] and peak 7 with a [M–H]^−^ ion at *m/z*: 177.01888 was identified as ferulic acid (C_9_H_5_O_4_^−^), peak 5 in turn as chlorogenic acid (C_16_H_17_O_9_^−^), and peaks 6 and 11 as feruloylquinic acid isomers (C_17_H_19_O_9_^−^). Peak 16 with a pseudomolecular ion at *m/z*: 529.13507 was identified as caffeoyl-feruloylquinic acid (C_26_H_25_O_12_^−^) and peak 17 with a pseudomolecular ion at *m/z*: 677.15009 as tricaffeoylquinic acid (C_34_H_29_O_15_^−^). Peak 29 with a [M–H]^−^ ion at *m/z*: 179.07108 was associated to dehidro p-methoxy-cumaric acid (C_10_H_11_O_3_^−^).

#### 3.8.3. Coumarins

Using co-elution procedures with already identified samples, we were able to identify peaks 10 and 13 as umbelliferone and scopoletin (**3**). Subject to further confirmation, peak 12 was identified as the dicoumarin euphorbetin because of its parent deprotonated ion at *m/z* 353.02919 [35,36] and peak 8 with a parent deprotonated molecule at *m/z* 515.08313 as its derivative euphorbetin glucoside (C_24_H_19_O_13_^−^). In the same manner, peak 2 with an anion at *m/z* 339.07217 was identified as esculin (esculetin-6-O-glucoside, C_15_H_15_O_9_^−^) and peak 4 as esculetin-6-O-(2-O’’ arabinosyl) glucoside (C_20_H_23_O_13_^−^), finally peak 3 was identified as fraxin (fraxetin-8-O-glucoside). The detailed metabolomic identification is explained below and the table with all data is depicted in the Table S1 (Supplementary Materials), plus examples and full MS spectra are shown in Figure S1 (Supplementary Materials).

#### 3.8.4. Flavonoids

Peak 20 was identified as isorhamnetin (C_16_H_11_O_7_^−^) because of the pseudomolecular ion at *m/z* 315.05090 [34]. Its identity was confirmed using co-injection with an authentic standard, and peak 18 and 19 as the flavonols kaempferol and quercetin, while peaks 23 and 24 as their derivatives 7-methoxykaempferol and (C_16_H_11_O_6_^−^) 7-methoxyquercetin (C_16_H_11_O_7_^−^), respectively. Peak 27 and 32 were identified as the polymethoxylated flavonoids 7,3′,5′-trimethoxymyricetin (C_18_H_15_O_8_^−^) and 3,7,3′-trimethoxyquercetin (C_18_H_15_O_7_^−^), respectively [37]. Through the spiking of experiments with authenticated samples, we were able to identify peaks 21, 26, 28, 30, and 31 as 5,4′-dihydroxy-7,3′-dimethoxyflavanone (1), 3,5,4′-trihydroxy-7,8,3′-trimethoxyflavone (6), 7-methoxy-eriodictyol, 5,4′-dihydroxy-3,7,8,3′-tetramethoxyflavone (7) and 5-hydroxy-7,3′,4′-trimethoxyflavanone (5), respectively.

#### 3.8.5. Tremetones

Peak 35 was identified as *p*-coumaroyloxytremetone (4) by spiking experiments with an authentic sample [38], and its isomer, peak 36 was tentatively identified as *m*-coumaroyloxytremetone (C_22_H_19_O_5_^−^), while peak 9 a more polar compound with an OH of difference with the previously mentioned compounds (Q-OT-ESI-MS at *m/z*: 379.11896) was identified as caffeoyloxytremetone (C_22_H_19_O_6_^−^) and finally peak 37 was identified as feruloyloxytremetone (C_23_H_21_O_6_^−^).

#### 3.8.6. Oxylipins

Peak 22 with a [M–H]^−^ ion at *m/z*: 327.21783 was identified as the dietary *Asparagus* oxylipins trihydroxyoctadecadienoic acid (C_18_H_31_O_5_^−^), while peak 25 with a [M–H]^−^ ion at *m/z*: 329.23367 was identified as trihydroxyoctadecaenoic acid (C_18_H_33_O_5_^−^) [39], and peak 34 as the oxylipin trihydroxydocosahexaenoic acid (C_22_H_31_O_5_^−^) [40].

## 4. Discussion

The hypotensive effects, and possible mechanisms, are involved in the ethnopharmacological uses of *P. quadrangularis* (crude and purified compounds) for treatment of cardiovascular complications. In folk medicine in northern Chile, *Parastrephia quadrangularis* concotions are mainly drunk as herbal teas, water infusions, or decoctions [3]. Therefore, scientific investigations and validation are useful for the preparation of nutritional supplements [41] or pharmacological preparations.

This study suggests that the hydroalcoholic extracts from Pq possess a negative inotropic effect, especially on its effects on calcium availability, and the possibility of reductions in peripheral resistance and blood pressure. The decrease of diastolic pressure could be through an alpha-adrenergic receptor blocker [42] and vasodilator effects, leading to a reduction in systemic vascular resistance.

Forty-three compounds including several tremetones, coumarins, and flavonoids were identified in the hydroalcoholic extract, and several of those compounds were isolated and tested regarding their hypotensive effects.

Results show that the negative inotropic effect and the reduction of heart rate observed could cause the drop of blood pressure and suggest a direct cardiac modulation by Pq extract [29], and that the effects of Pq in reducing vascular contractile response on endothelium-intact rings occurs in a dose-dependent manner. In fact, the absence of the endothelium or the inhibition of the NOS with L-NAME significantly reduced the Pq-induced vasodilation in aorta. Traditionally, vasoactive substances cause vasodilation either by stimulating the NO/sGC pathway, activating the potassium channels, or blocking the Cav1.2 channels [43]. However, the inhibition of guanylate cyclase soluble (sGC) with ODQ did not blunt the Pq-induced vasodilation, suggesting another mechanism involved, different to the NO/sGC pathway.

Schinzari et al. [44] reported that potassium channel activation in vascular smooth muscles leads to the hypolarization of the vascular membrane, and an increase in vasodilation. The preincubation with BaCl_2_, glibenclamide, and TEA significantly diminished the Pq-induced relaxation.

Another possibility may be that Pq extract produced vasodilation through a modulation of influx of calcium from extracellular sources through Ca_V_1.2 [45]. This observation was confirmed by subsequent experiments. First, the preincubation with Pq reduced the contractile response mediated by the membrane depolarization with KCl and pharmacological stimulation with PE. Secondly, the effects of preincubation with Pq and nimodipine (nonselective blocker of L-type voltage-gated Ca^2+^ channels) on PE induced contractile responses to the aortic rings were alike. Thirdly, there was a decrease in the A7r5 vascular smooth muscle cell line cytosolic calcium with PE following incubation with Pq, which was also observed with Bay K8644 that acts through Cav1.2 channels on intact aortic rings.

To evaluate the vasodilation effect of the seven pure isolated compounds, a screening test was conducted in intact aortic rings of rats. We found that the flavonoids: 5,3′,4′-trihydroxy-7-methoxyflavanone (7-methoxy-eriodictyol) (2), 3,5,4′-trihydroxy-7,8,3′-trimethoxyflavone (6) and 5,4′-dihydroxy-3,7,8,3′-tetramethoxyflavone (7) decreased the PE induced vascular contractions just like Pq extract and ACh. These activities could be due to some synergism (presence of phenolics), or in part by blocking or antagonizing calcium Cav1.2 channels in aortic rings.

These findings strongly suggest that the hypotensive activity described above could be explained by a decrease in peripheral resistance. Notwithstanding the fact that vessels like the aortas are conductance vessels, and play little roles in peripheral resistance, the vascular reactivity properties of the extract shows that it could reduce peripheral resistance through a reduction in vascular myogenic tone. This is in agreement with our results and is complementary to the negative inotropic effects seen in the cardiac musculature of the heart.

The extract Pq exhibited moderate-high antioxidant properties, with phenolic and flavonoid constituents, and similar vascular abilities following pre-contraction with agonists like Bay K8644 (an agonist of Ca_V_1.2 channels) and PE. These data agree with previous studies of different species of *Parastrephia* (*P. lepidophylla, P. lucida,* and *P. phyliciformis* (Meyen) Cabrera) [13].

Three isolated flavonoids caused a reduction of the contractile vascular response to influx of extracellular Ca^2+^ and was likely mediated by blockage of Cav1.2 channels. In addition, Pq extract reduced the contractile response to PE in a similar way at nimodipine (a nonselective blocker of L-type voltage-gated Ca^2+^ channels).

Vascular relaxation observed by the extract and three flavonoids isolated from Pq, which was similar to ACh-induced endothelial vasodilation via endothelial NO, strongly suggest that antioxidant activity of Pq and its metabolites may be involved. We hypothesize that three isolated flavonoids would increase the bioavailability of endothelial NO, leading to endothelial vasodilation. In contrast to flavonoids 2, 6, and 7 isolated from Pq, quercetin, a standard flavonoid with a high antioxidant activity, did not cause a significant vasodilation. This result would indicate that quercetin exerts a cardio-protector effect by other pathways, such as preventing the lipopolysaccharide-induced oxidative stress, or reducing lipid peroxidation and protein oxidation [46].

## 5. Conclusions

In conclusion, *P. quadrangularis* demonstrated hypotensive ability in normotensive animals; the mechanisms involved include an increase in vasodilation response, a decrease in heart rate, and cardiac contractility via inotropic effects. The mechanisms associated with these effects include endothelium-dependent vasodilation by NO and independent mechanisms, an activation of the potassium channels, and a decrease in cytosolic calcium.

## Figures and Tables

**Table 1:** Effect of *P. quadrangularis* (Pq) on mean arterial pressure (MAP) administrated intravenously in normotensive rats.

Blood Pressure	Control	10 mg/kg Pq	20 mg/kg Pq	40 mg/kg Pq	80 mg/kg Pq
MAP, mmHg	128 ± 4	103 ± 2	94 ± 8 **	82 ± 6 ***	55 ± 3 ***

Values are mean ± standard error of the mean of 5 experiments in mmHg. ** *p* < 0.01 and *** *p* < 0.001 vs. control.

**Table 2:** Effect of *P. quadrangularis* (Pq) on systolic blood pressure (SBP), diastolic blood pressure (DBP) and pulse pressure (PP) of normotensive rats.

Blood Pressure	Control	10 mg/kg Pq	20 mg/kg Pq	40 mg/kg Pq
SBP, mmHg	111 ± 6	107 ± 2	103 ± 2	99 ± 2
DBP, mmHg	75 ± 6	73 ± 1	66 ± 3	49 ± 1 **
PP, mmHg	36 ± 5	35 ± 3	37 ± 3	50 ± 2 *

Values are mean ± standard error of the mean of 5 experiments in mmHg. * *p* < 0.05; ** *p* < 0.01 vs. control.

**Table 3:** Effect of *P. quadrangularis* (Pq) on the vascular response to different vasoactive substances in rat aorta.

Drugs	IC_50_ (µg/mL)
Control	122 ± 1
Endo-denuded	272 ± 2 ***
L-NAME	903 ± 2 ***
ODQ	104 ± 2 ***
BaCl_2_	275 ± 4 ***
Glibenclamide	122 ± 1
TEA	410 ± 2 ***

IC_50_ represent the half maximal inhibitory concentration. The values are mean ± standard error of the mean (SEM) and represents the mean of 5 independent experiments. Statistically significant difference *** *p* < 0.001 vs. control; L-NAME: Nω-nitro-L-arginine methyl ester; ODQ: 1H-(1,2,4)oxadiazolo[4,3-a]quinoxalin-1-one; TEA: tetraethyl ammonium.

**Table 4:** Effect of *P. quadrangularis* (Pq) and nimodipine on the vascular response to KCl and phenylephrine (PE), in rat aorta.

Drugs	EC_50_
KCl (mM)	
Control	21.0 ± 1
Pq	24.0 ± 4
Nimodipine	n.c.
PE (nM)	
Control	29.9 ± 1
Pq	95.0 ± 2 **
Nimodipine	89.8 ± 1 *

EC_50_ represents the half maximal effective concentration. The values are mean ± SEM represents the mean of 5 independent experiments. Statistically significant difference * *p* < 0.05, ** *p* < 0.01 vs. control, *n* = 5.

**Table 5:** Value of hydroalcoholic extract of *Parastrephia quadrangularis* (Pq) for various antioxidant systems.

Antioxidant Assay	Pq	Trolox	Ascorbic Acid
Total phenolics **^a^**	482 ± 19	-	-
Total flavonoids **^b^**	140 ± 4	-	-
FRAP **^c^**	760 ± 12	-	-
ABTS **^d^**	127 ± 5 *	77 ± 2	-
DPPH **^d^**	201 ± 4 *	61 ± 3	-
NO **^d^**	498 ± 5 *	-	48 ± 1
Molybdate **^d^**	115 ± 4 *	-	23 ± 1
Hexacyanoferrate (III) **^d^**	73 ± 2 *	-	19 ± 3

(**a**) Expressed in mg gallic acid equivalent/g dry extract, (**b**) expressed in mg quercetin equivalent/g dry extract, (**c**) expressed in mg trolox equivalent/g dry extract, and (**d**) IC_50_ expressed in μg/mL extract. All values were expressed as means ± SEM (*n* = 4). * *p* < 0.05 vs. Pq. FRAP: ferric reducing/antioxidant power; ABTS: 2,2′-azino-bis(3-ethylbenzothiazoline-6-sulfonic acid; DPPH: 2,2-diphenyl-1-picryl-hydrazyl-hydrate; NO: nitric oxide.

## Data Availability

The datasets generated during and analyzed during the current study are available from the corresponding author on reasonable request.

## Supplementary Materials

The following are available online at https://www.mdpi.com/2076-3921/8/12/591/s1, Supplementary File 1: Methods, Table S1: High resolution UHPLC PDA-Q orbitrap identification of metabolites in the hydroalcoholic extract of Parastrephia quadrangularis (Pq), Figure S1: Structures and full MS spectra of compounds 5, 8, 9, 16, 36, and 37.
