# Supplementary material for: Polyphenolic Composition and Hypotensive Effects of Parastrephia quadrangularis (Meyen) Cabrera in Rat
Source: Antioxidants (Basel). 2019 Nov 27;8(12):591. doi: 10.3390/antiox8120591 (PMC6943605; doi:10.3390/antiox8120591)
Supplement: Supplementary file 1 [file antioxidants-08-00591-s001.pdf]

Supplementary materials

Methods

1. *Phenolic content (TPC) estimation.*

The TPC method was based on the Folin–Ciocalteu method. A total of 20 µL of the diluted extract (500 µg/mL) were mixed with 100 µL of 10% (vol/vol) of Folin–Ciocalteu reagent and shaken. After 5 mins, 75 µL of $NaCO_3$ (700 mM) was added, and absorbance measured at 765 nm using a microplate reader after 1 hour at room temperature. Gallic acid dilutions (0-1000 µg/mL) were used as standards for calibration. Data from these multiple experiments were presented as milligram of gallic acid equivalent per gram of dry extract.

2. *Total flavonoid content (TFC) estimation.*

Briefly, a mixture of 50 µL extracts, 25 µL of aluminum chloride (10%), 80 µL of methanol, and 25 µL of 1 M potassium acetate were placed in a micro-plate, and absorbance read at 510 nm after incubation for 30 min. Analyses were carried out in quadruplicate and the results were expressed as mg quercetin equivalent per gram of dry extract.

3. *Ferric reducing antioxidant power (FRAP) assay.*

The FRAP reagent composed of 300 mM buffer acetate at pH 3.6, 2,4,6-tris-(2-pyridyl)-s-triazine 10 mM (TPTZ) in hydrochloric acid 40 mM and $FeCl_3.6H_2O$ aqueous solution 20 mM in the ratio of 10:1:1 (v/v). 70 µL FRAP solution was mixed with 10 µL extract solution at 500 µg/mL, absorbance was read at 593 nm, and compared with 0 - 500 µg/m trolox solution (standard). Results were presented as mg of trolox/g of dry extract. Experiments were performed in multiples of two.

4. *Determination of ABTS radical-scavenging activity*

Briefly, a mixture of 2.5 mM $K_2S_2O_8$, methanol and ABTS in phosphate buffer saline at pH 7.4 was corrected for absorbance at 734 nm, and kept in the dark at 22°C. A mixture of 180 µL of ABTS in PBS and 20 µL of PBS was used as the blank solution. The radical scavenging properties were measured using Trolox as a standard, calculated as concentration required to scavenge 50% of ABTS radicals, and expressed as IC_{50} (µg/mL). Experiments were performed in multiples of two

5. *DPPH Radical Scavenging Activity Assay*

Briefly, a 0.2 mM solution of 1,1-diphenyl-2-picrylhydrazyl (DPPH) in methanol was prepared, and 70 µL of this solution was added to 20 µL of extract (0-1000 µg/mL). Trolox in concentrations of 0-1000 µg/mL was used as a standard reference antioxidant. Discoloration of the reaction mixture was measured at 517 nm after incubation for 30 min. The results were expressed as IC_{50} (concentration of extract or standard in µg/mL required to inhibit 50% of DPPH radical present in solution). Analyses were carried out in quadruplicate.

6. *Determination of Nitric oxide-scavenging activity*

The scavenging effects of extracts on nitric oxide (NO) were carried out by the following method. A total of 40 µL of various concentrations of each positive control or extract dissolved in 50 mM of phosphate buffered saline (PBS; pH 7.4) was added to a 96 well microplate containing 20 µL of sodium nitroprusside (SNP; 25 mM) in PBS. The mixture was incubated at 37 ºC for 2 h under normal light exposure. Control set without the test compounds but with 40 µL of PBS was conducted in an identical manner. The mixture was diluted with 50 µL of Griess reagent (0.5% sulphanilamide and 0.05% naphthylethylenediamine dihydrochloride in 2.5% H_3PO_4) and the absorbance was measured at 570 nm. To evaluate the NO scavenging effect of extract, the NO scavenger ascorbic acid was used as positive control for comparison. The NO-scavenging effect was expressed as an IC_{50} value (concentration in µg/mL required to inhibit NO formation by 50%), following the construction of a standard curve for sodium nitrate.

7. *Phosphomolybdate assay*

The antioxidant capacity was evaluated with a quantitative method of phosphomolybdate reagent. Exact 10 µL of the extract (500 µg/mL) was mixed with the phosphomolybdenum reagent (100 µL, composed of sulphuric acid 0.6 M, sodium phosphate, 28 mM and ammonium molybdate, 4 mM). The tubes with the mixture were then placed in a dry bath heat blocks at 95 ºC for 90 minutes, then cooled and the measurement was performed at 765 nm. Antioxidant activity is expressed as mg of ascorbic acid/gram of extract, following the construction of a standard curve. Analyses were carried out in quadruplicate.

8. Reducing power determination

An exact aliquot of 20 μL (500 μg/mL) of the extracts was mixed with 500 μL of phosphate buffer (0.2 M, pH 6.6) and 500 μL of 1% (w/v) potassium ferricyanide. The mixture was set at 50 °C incubated for 20 min. Adding 500 μL of 10% (w/v) trichloroacetic acid to the mixture stopped the reaction. Mixture was then centrifuged (3,000 rpm) for 10 min. 50 μL of supernatant was then collected, mixed with 10 μL ferric chloride (0.1% w/v at a ratio of 1:1:0.2 v.v.v.v) and 50 μL of distilled water. The concentration of ferric–ferrocyanide was determined at 700 nm using ascorbic acid as standard. Analysis were carried out in quadruplicate.

Table. S1. High resolution UHPLC PDA-Q orbitrap identification of metabolites in the hydroalcoholic extract of *Parastrephia quadrangularis* (Pq)

Peak #	**Retention time (min.)**	**UV max**	**Tentative identification**	**Elemental composition [M-H]**	**Theoretical mass (*m/z*)**	**Measured mass (*m/z*)**	**Accuracy (δppm)**	**MS^n ions (δppm)**
1	1.29	-	Gluconic acid	$C_6H_{11}O_7^-$	195.05103	195.05057	-18.72	
2	1.61	305-350	Esculin (Esculetin-6-O-glucoside)	$C_{15}H_{15}O_9^-$	339.07216	339.07217	0.02	
3	8.76	305-348	Fraxin (Fraxetin-8-O-glucoside)	$C_{16}H_{17}O_{10}^-$	369.08272	369.08267	-0.13	161.02377 $C_9H_5O_3^-$ (umbelliferone)
4	9.17	299-325	Esculetin-6-O-(2-O´´ arabinosyl) glucoside	$C_{20}H_{23}O_{13}^-$	471.11441	471.11438	-0.06	177.01869 $C_9H_5O_4^-$ (esculetin)
5	9.37	255-325	Chlorogenic acid	$C_{16}H_{17}O_9^-$	353.08781	353.08786	0.14	191.05556 $C_7H_{11}O_6^-$ (quinic acid)
6	9.57	255-325	Feruloylquinic acid	$C_{17}H_{19}O_9^-$	367.10346	367.10352	0.16	134.03656 $C_8H_6O_2^-$(decarboxylated ferulic acid) 193.05013 $C_{10}H_9O_4^-$(ferulic acid)
7	9.75	255-310	Ferulic acid	$C_9H_5O_4^-$	177.01933	177.01888	-2.5	133.02879 $C_8H_5O_2^-$(decarboxylated ferulic acid)
8	9.90	299-345	Euphorbetin glucoside	$C_{24}H_{19}O_{13}^-$	515.08311	515.08313	0.03	177.01881 $C_9H_5O_4^-$ (esculetin)
9	10.16	325	Caffeoyloxytremetone	$C_{22}H_{19}O_6^-$	379.11871	379.11896	0.65	117.03378 $C_8H_5O^-$; 145.02873 $C_9H_5O_2^-$
10	10.35	295-345	Scopoletin (**3**)	$C_{10}H_7O_4^-$	191.03498	191.03448	-2.61	

11	10.48	255-325	Feruloylquinic acid	$C_{17}H_{19}O_9^-$	367.10346	367.10361	0.40	173.04492 $C_7H_9O_5^-$ (quinic acid); 134.03665 $C_8H_6O_2^-$
12	10.76	285-345	Euphorbetin	$C_{18}H_9O_8^-$	353.03046	353.03049	0.08	
13	11.37	285-345	Umbelliferone	$C_9H_5O_3^-$	161.02442	161.02386	-3.4	
14	11.71	255-325	Dicaffeoylquinic acid	$C_{25}H_{23}O_{12}^-$	515.11950	515.11932	0.34	191.05563 $C_7H_{11}O_6^-$ (quinic acid); 135.04437 $C_8H_7O_2^-$
15	11.90	255-325	Dicaffeoylquinic acid	$C_{25}H_{23}O_{12}^-$	515.11950	515.11938	-0.23	173.04494 $C_7H_9O_5^-$ (shikimic acid); 135.04437 $C_8H_7O_2^-$
16	12.63	310	Caffeoyl-feruloylquinic acid	$C_{26}H_{25}O_{12}^-$	529.13515	529.13507	-0.15	173.04503 $C_7H_9O_5^-$ (shikimic acid); 134.03654 $C_8H_6O_2^-$
17	13.55	-	Tricaffeoylquinic acid	$C_{34}H_{29}O_{15}^-$	677.15119	677.15009	-1.62	173.04495 $C_7H_9O_5^-$ (shikimic acid); 135.04442 $C_8H_7O_2^-$
18	13.86	255-365	Kaempferol	$C_{34}H_{29}O_{15}^-$	285.04046	285.04053	0.24	133.02882 $C_8H_5O_2^-$
19	14.06	254-354	Quercetin	$C_{15}H_9O_7^-$	301.03538	301.03546	0.26	107.01305 $C_6H_3O_2^-$; 151.00296 $C_7H_3O_4^-$
20	14.78	254-354	Isorhamnetin	$C_{16}H_{11}O_7^-$	315.05103	315.05112	0.28	135.04446 $C_8H_7O_2^-$; 161.02396 $C_9H_5O_3^-$
21	15.20	281	5,4’-dihydroxy-7,3’-dimethoxyflavanone (**1**)	$C_{17}H_{15}O_6^-$	315.08741	315.08151	-18.7	173.04498 $C_7H_9O_5^-$ (shikimic acid); 135.04425 $C_8H_7O_2^-$
22	16.28	218	Trihydroxyoctadecadienoic acid	$C_{18}H_{31}O_5^-$	327.21770	327.21783	0.39	
23	17.13	256-366	7-Methoxykaempferol	$C_{16}H_{11}O_6^-$	299.05611	299.05624	0.43	
24	17.83	254-354	7-Methoxyquercetin	$C_{16}H_{11}O_7^-$	315.05103	315.05109	0.190	
25	19.00	225	Trihydroxyoctadecaenoic acid	$C_{18}H_{33}O_5^-$	329.23335	329.23367	0.97	
26	18.46	255-354	5-hydroxy-7,4’,3’-trimethoxyflavanone (**5**)	$C_{18}H_{15}O_8^-$	359.07724	359.07745	0.58	
27	18.99	254-354	7, 3’, 5’-trimethoxymyricetin	$C_{18}H_{15}O_8^-$	359.07724	359.07727	0.08	

28	19.19	283	5,3’4’-trihydroxy-7-methoxyflavanone (7-methoxy-eriodictyol) (**2**)	$C_{16}H_{13}O_6^-$	301.07176	301.07187	0.36	135.04440 $C_8H_7O_2^-$
29	19.41	275-325	Dehidro *p*-methoxy-coumaric acid	$C_{10}H_{11}O_3^-$	179.07137	179.07108	-1.61	133.02882 $C_8H_5O_2^-$
30	19.69	254-354	3,5,4’-trihydroxy-7,8,3’-trimethoxyflavone (**6**)	$C_{19}H_{17}O_8^-$	373.09289	373.09286	-0.08	
31	19.84	281	5,4’-dihydroxy-3,7,8,3’-tetramethoxyflavone (**7**)	$C_{18}H_{17}O_6^-$	329.10306	329.10321	0.45	
32	20.07	254-354	7,3,3'-trimethoxyquercetin	$C_{18}H_{15}O_7^-$	343.08233	343.08258	0.72	
33	20.48	254-354	5, 4’-dihydroxy-3’7, 8-trimethoxyflavone	$C_{17}H_{13}O_8^-$	345.09798	345.09180	-17.90	
34	20.79	215	Trihydroxydocosahexaenoic acid	$C_{22}H_{31}O_5^-$	375.21770	375.21771	0.02	
35	20.89	275-310	p-Coumaroyltremetone (**4**)	$C_{22}H_{19}O_5^-$	363.12380	363.12387	0.19	117.03374 $C_8H_5O^-$; 145.02869 $C_9H_5O_2^-$
36	21.12	275-324	m-Coumaroyoxyltremetone	$C_{22}H_{19}O_5^-$	363.12380	363.12384	0.11	117.03370 $C_8H_5O^-$; 145.02867 $C_9H_5O_2^-$
37	21.47	275-324	Feruloyloxytremetone	$C_{23}H_{21}O_6^-$	393.13436	393.13452	0.40	117.03378 $C_8H_5O^-$; 145.02873 $C_9H_5O_2^-$

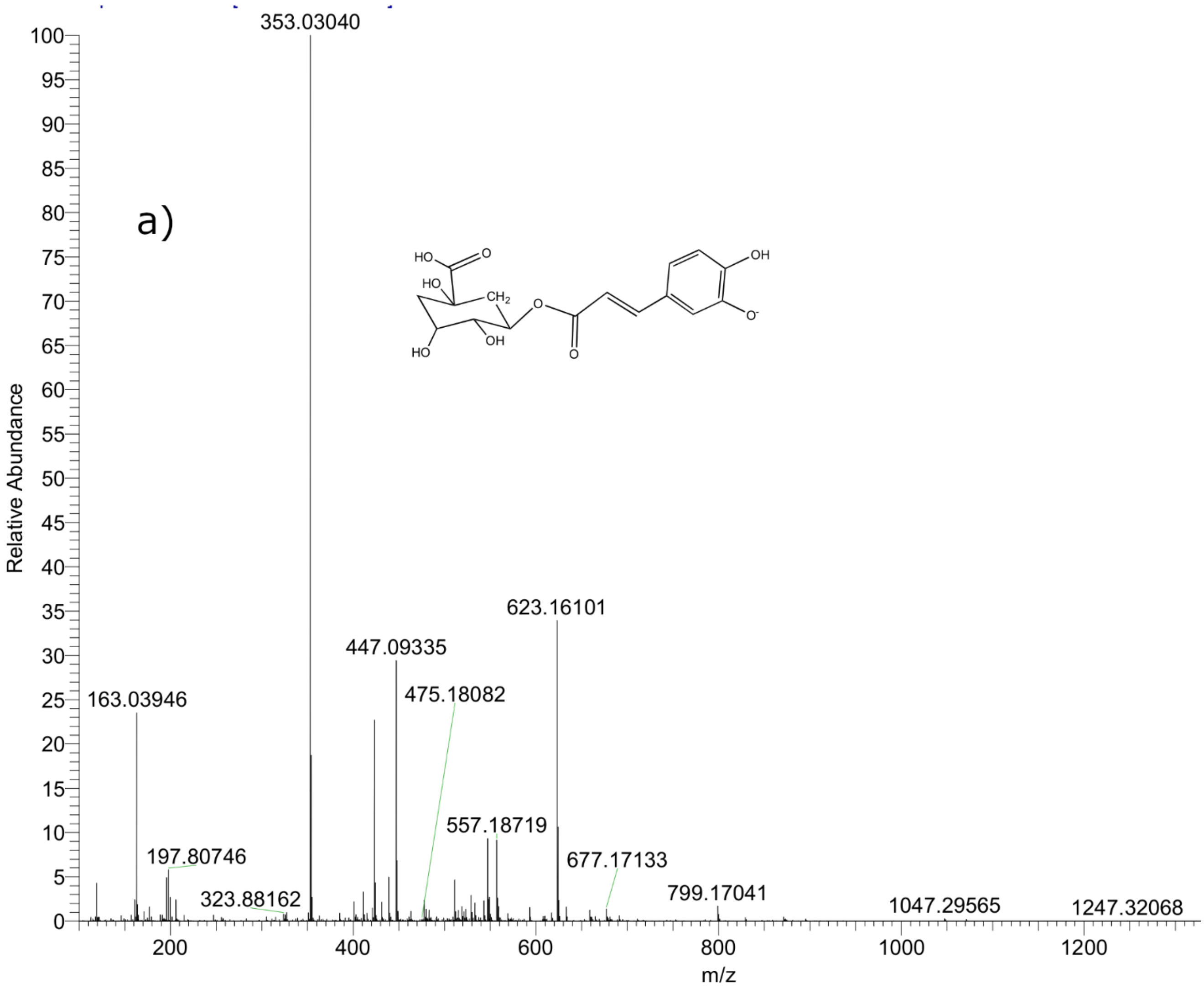
a)
353.03040
623.16101
447.09335
475.18082
163.03946
557.18719
197.80746
677.17133
323.88162
799.17041
1047.29565
1247.32068
Relative Abundance
m/z
HO
O
HO
CH2
O
OH
O-
HO
OH
O

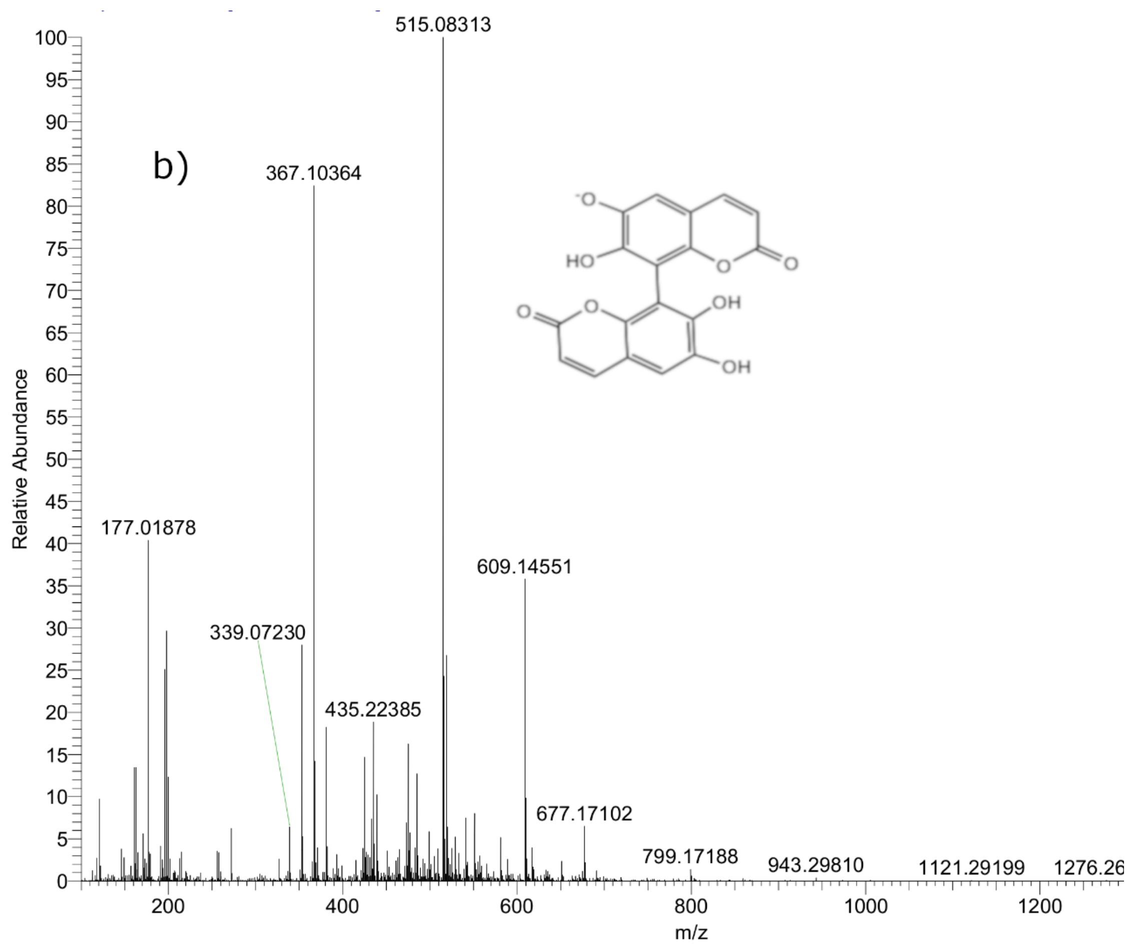
b)
515.08313
367.10364
177.01878
609.14551
339.07230
435.22385
677.17102
799.17188
943.29810
1121.29199
1276.26
HO
OH
OH
Relative Abundance
m/z

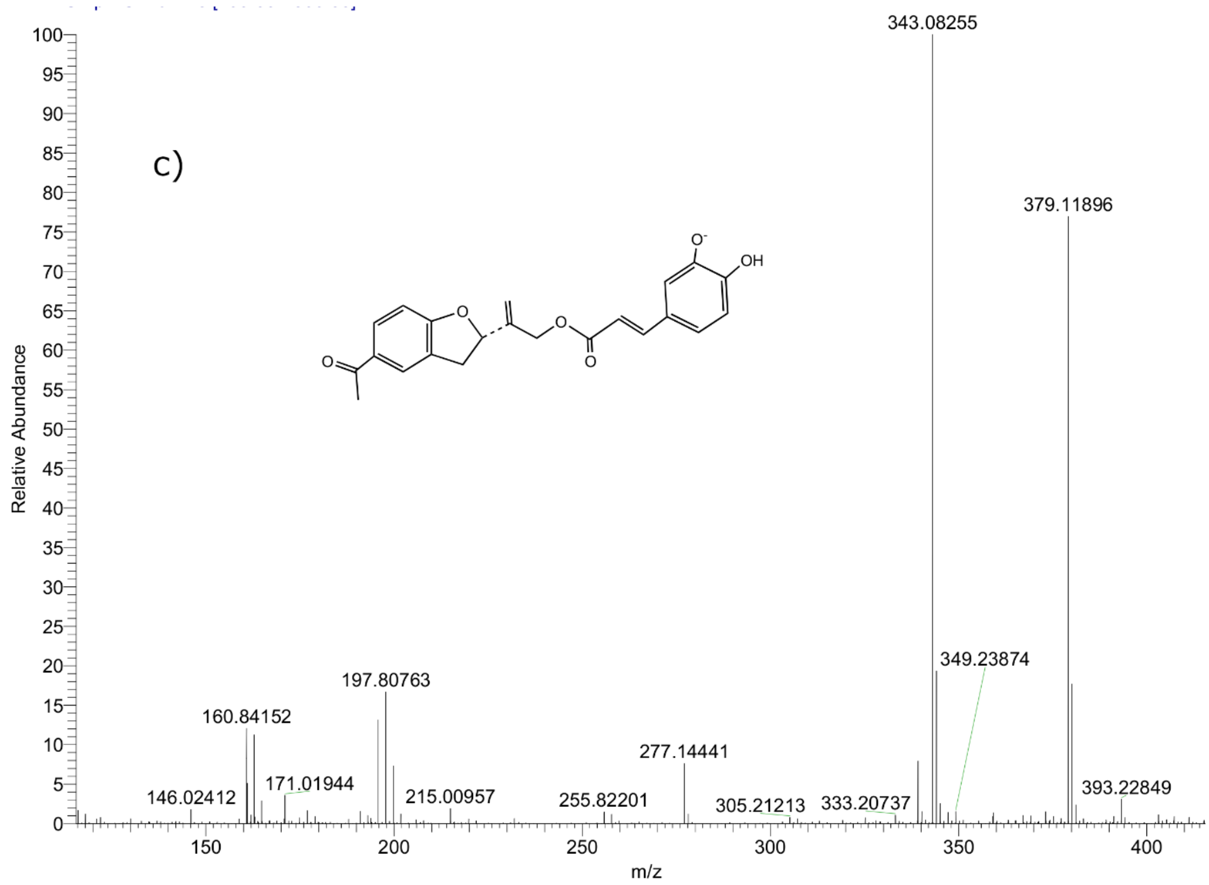
c)
343.08255
379.11896
349.23874
197.80763
160.84152
171.01944
146.02412
215.00957
255.82201
277.14441
305.21213
333.20737
393.22849
OH
O
Relative Abundance
m/z

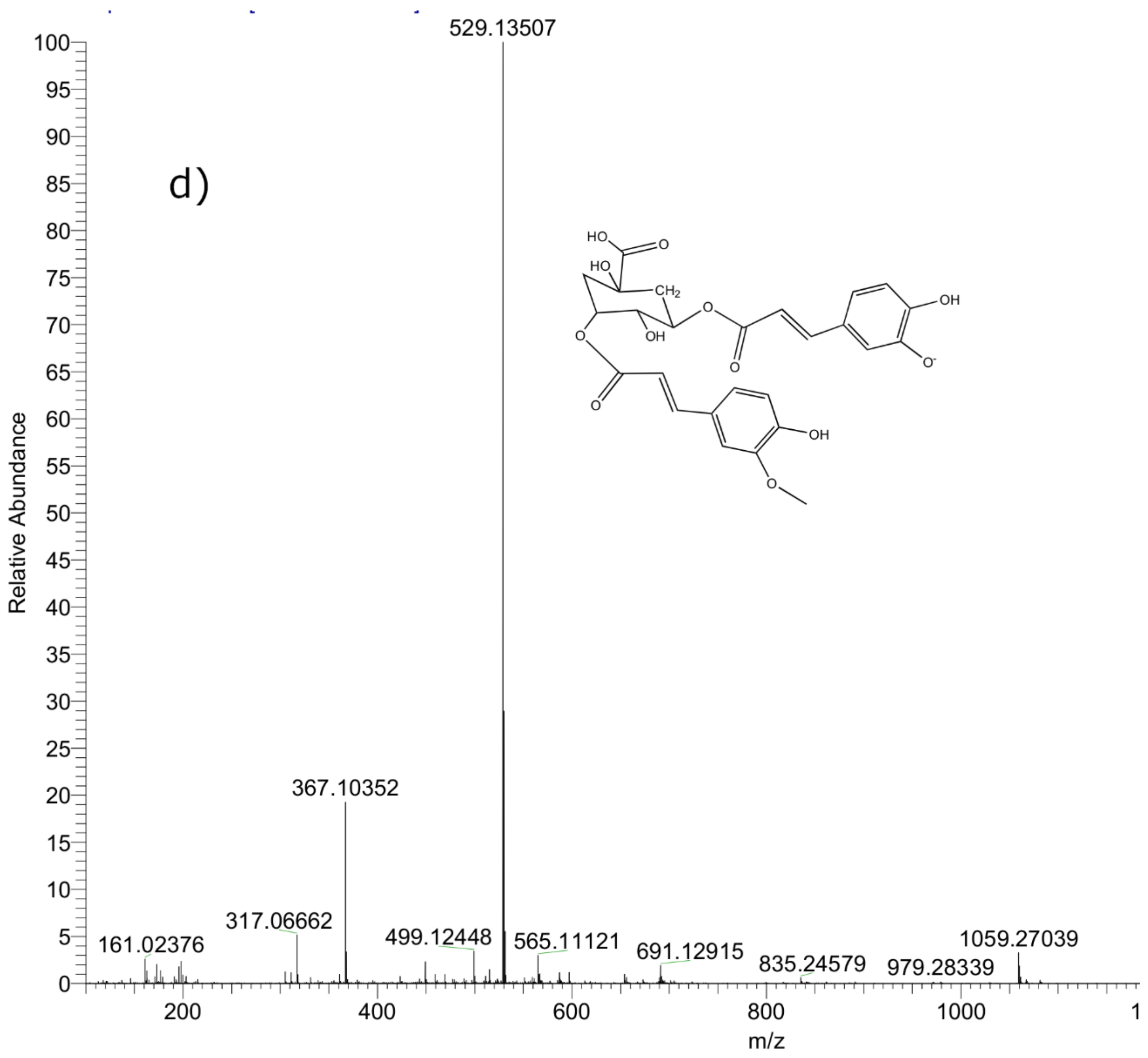

d)
529.13507
367.10352
317.06662
161.02376
499.12448
565.11121
691.12915
835.24579
979.28339
1059.27039
Relative Abundance
m/z
HO
O
HO
CH2
OH
OH
O-
O
OH

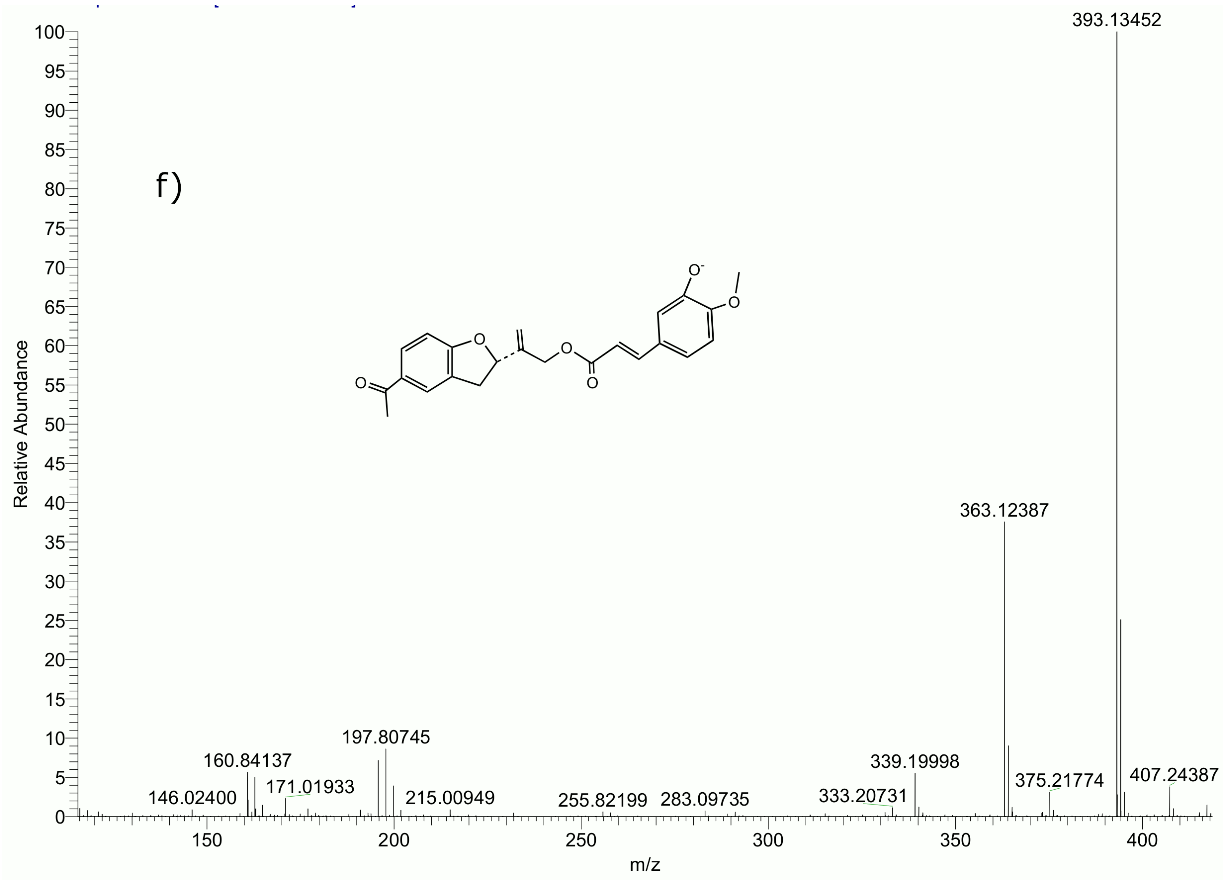

Figure S1. Structures and full MS spectra of compounds 5, 8, 9, 16, 36 and 37.
